# Promoting and growing your podcasts and ensuring sustainability and consistency in your approach

**DOI:** 10.1093/eurpub/ckac129.024

**Published:** 2022-10-25

**Authors:** J Rachadell

**Affiliations:** 1 EUPHA-DH; 2 Public Health Unit, ACES Lisboa Ocidental e Oeiras, Lisbon, Portugal; 3 Institute for Evidence-based Health, Lisbon, Portugal

## Abstract

The main challenges after getting started with a podcast will be growth and sustainability. This presentation will focus on promotion tactics for digital health podcasts, including the pros and cons of different hosting platforms and the use of social and academic networks for promotion. The importance of sustainability and consistency for growth will also be discussed, including some practical tips on productivity and team management for content producers.

